# Gender perspective on the association between liver enzyme markers and non-alcoholic fatty liver disease: insights from the general population

**DOI:** 10.3389/fendo.2023.1302322

**Published:** 2023-12-06

**Authors:** Jiajun Qiu, Maobin Kuang, Shiming He, Changhui Yu, Chao Wang, Xin Huang, Guotai Sheng, Yang Zou

**Affiliations:** ^1^ Department of Internal Medicine, Medical College of Nanchang University, Jiangxi Provincial People’s Hospital, Nanchang, Jiangxi, China; ^2^ Jiangxi Cardiovascular Research Institute, Jiangxi Provincial People’s Hospital, The First Affiliated Hospital of Nanchang Medical College, Nanchang, Jiangxi, China; ^3^ Jiangxi Provincial Geriatric Hospital, Jiangxi Provincial People’s Hospital, The First Affiliated Hospital of Nanchang Medical College, Nanchang, Jiangxi, China

**Keywords:** liver enzyme marker, non-alcoholic fatty liver disease, alanine aminotransferase, ALT, fatty liver

## Abstract

**Objective:**

Every distinct liver enzyme biomarker exhibits a strong correlation with non-alcoholic fatty liver disease (NAFLD). This study aims to comprehensively analyze and compare the associations of alanine aminotransferase (ALT), aspartate aminotransferase (AST), and gamma-glutamyl transferase (GGT) with NAFLD from a gender perspective.

**Methods:**

This study was conducted on 6,840 females and 7,411 males from the NAGALA cohort. Multivariable logistic regression analysis was used to compare the associations between liver enzyme markers and NAFLD in both genders, recording the corresponding adjusted odds ratios (ORs) and 95% confidence intervals (CIs). Receiver operating characteristic (ROC) curves were used to evaluate the accuracy of individual liver enzyme markers and different combinations of them in identifying NAFLD.

**Results:**

Liver enzyme markers ALT, AST, and GGT were all independently associated with NAFLD and exhibited significant gender differences (All *P*-interaction<0.05). In both genders, ALT exhibited the most significant association with NAFLD, with adjusted standardized ORs of 2.19 (95% CI: 2.01-2.39) in males and 1.60 (95% CI: 1.35-1.89) in females. Additionally, ROC analysis showed that ALT had significantly higher accuracy in identifying NAFLD than AST and GGT in both genders (Delong *P*-value < 0.05), and the accuracy of ALT in identifying NAFLD in males was higher than that in females [Area under the ROC curve (AUC): male 0.79, female 0.77]. Furthermore, out of the various combinations of liver enzymes, ALT+GGT showed the highest accuracy in identifying NAFLD in both genders, with AUCs of 0.77 (95% CI: 0.75-0.79) in females and 0.79 (95% CI: 0.78-0.81) in males.

**Conclusion:**

Our study revealed significant gender differences in the associations of the three commonly used liver enzyme markers with NAFLD. In both genders, the use of ALT alone may be the simplest and most effective tool for screening NAFLD, especially in males.

## Introduction

NAFLD is a chronic liver condition characterized by the presence of excessive fat deposits in liver cells without significant alcohol consumption (1). Epidemiological studies have shown that the incidence of NAFLD is increasing globally in parallel with the rising obesity rates (the latest estimate of the global incidence is approximately 46.13/1000 person-years) (2), making it the most common chronic liver disease (3, 4). While NAFLD itself may not directly cause mortality, it is a significant contributor to other major health problems such as liver cancer, diabetes, and cardiovascular diseases, which contribute to increased mortality rates and impose a substantial economic and disease burden on countries worldwide. Given its extensive impact on multiple organ systems and high incidence, early screening and prevention are crucial in reducing the occurrence of NAFLD and decreasing the burden of the disease (5). It is worth noting that despite the significant gender differences in the occurrence, progression, and incidence of NAFLD (6, 7), the impact of gender on NAFLD is often overlooked (8). Therefore, in the context of the modern trend toward precision medicine, quantifying gender differences and increasing knowledge of NAFLD can help develop gender-specific personalized screening and prevention strategies to manage the increasing disease burden of NAFLD worldwide.

The diagnosis and risk stratification of NAFLD rely on clinical, biochemical, imaging, and histological findings (9). Liver biopsy is the gold standard for confirming NAFLD, but its high cost and invasive nature contradict the Helsinki Declaration and are less frequently used in primary care settings (10). Therefore, in clinical practice, non-invasive methods are preferred for screening and monitoring NAFLD. Currently, Doppler ultrasonography is the main diagnostic tool for NAFLD in a clinical setting; however, access to high-quality ultrasound examinations is limited in some developing countries with a heavy burden of NAFLD (11, 12). In light of this, the World Gastroenterology Organization has provided recommendations for a tiered screening approach in its guidelines (13), in which liver function enzymes (including ALT, AST, and GGT) are suggested as initial screening measures for NAFLD.

Liver enzymes are widely distributed in the liver and biliary system, with ALT mainly present in the liver cytoplasm, AST predominantly found in liver cell mitochondria, and GGT originating primarily from the liver and biliary system. Studies have shown that ectopic fat accumulation in the liver, which leads to hepatic steatosis, result in liver cell damage and inflammation occur, and, in turn, cause liver enzymes (ALT, AST, GGT) to be released into the bloodstream (14–17). Although elevated liver enzymes are not exclusive to NAFLD, NAFLD is the most common cause of abnormal liver function (18). It has been reported that approximately 80% of cases with abnormal liver function in the American are attributed to NAFLD (19). Therefore, liver enzymes are widely used as a cost-effective screening tool for NAFLD in primary healthcare settings worldwide. The association between liver enzymes and NAFLD is well-established, and in most studies, liver enzymes are used as surrogate markers to identify the disease (20, 21). However, there are significant differences in the occurrence, progression, and plasma levels of liver enzymes between males and females (8). Previous research has shown that levels of ALT, AST, and GGT were higher in males than in females, which may be related to differences in basal metabolic rate and liver enzyme activity between the genders (22). Additionally, androgens promote the development of NAFLD (23, 24), while estrogens have a protective effect in females (25, 26). Furthermore, evidence from randomized controlled trials indicated that estrogens can significantly lower liver enzyme levels in patients with type 2 diabetes (27). These findings collectively emphasize that genders may influence the relationship between liver enzymes and NAFLD. Therefore, in the screening and diagnosis of NAFLD, liver enzyme indicators need to be quantified, analyzed, and interpreted according to the genders. However, although ALT is widely regarded as the most sensitive marker of liver injury in the general population (28, 29), there is currently a lack of evidence regarding which liver enzyme indicator is most suitable for screening NAFLD. Furthermore, it remains unclear whether there exists a specific gender-specific combination of liver enzymes that can significantly improve the screening efficiency of NAFLD in both genders. Therefore, this study aims to comprehensively analyze and compare the associations between ALT, AST, GGT, and NAFLD in both genders using cross-sectional data from a large sample of the general population, explore the most suitable combination of liver enzymes for screening NAFLD in both genders, and provide new insights for public health agencies to develop gender-specific screening strategies, thereby improving the accuracy and efficiency of NAFLD screening.

## Methods

### Study population

The study samples were obtained from the NAGALA cohort, a large prospective cohort of the general population in Japan. The NAGALA cohort is a longitudinal study conducted at the Murakami Memorial Hospital of Gifu, which aimed at assessing the relationship between chronic diseases and their risk factors by utilizing data from various anthropometric and biochemical measurements taken during health examinations of individuals at the hospital. The available data and materials for the study have been uploaded by Professor Okamura to the Dryad public database for online sharing (30). According to the terms of service of the Dryad database, we can perform secondary analysis based on new research hypotheses after indicating the source of the data, which is not in violation of the author’s rights. In the current study, we aimed to further analyze the associations between liver enzymes and NAFLD from a gender perspective using this large cohort dataset. We also aimed to explore the optimal combination of liver enzymes for disease screening, providing evidence for the development of more accurate NAFLD screening and diagnostic protocols while reducing healthcare resource expenditure.

The detailed research design, methods, and major findings of the NAGALA cohort have been previously published (31). In brief, the NAGALA cohort started in 1994 and has been followed up to the present. The participants were recruited from the general population undergoing health examinations at the Murakami Memorial Hospital of Gifu. They underwent questionnaire surveys, general physical measurements, laboratory blood tests, and liver ultrasound examinations at the time of enrollment, providing data on demographic information, health status, lifestyle habits, physical measurement parameters, as well as laboratory tests and examinations. The NAGALA project had previously been approved by the Ethics Committee of the Murakami Memorial Hospital of Gifu, and complied with the Helsinki Declaration; and all participants provided written informed consent. In accordance with national regulations, the study author’s institutional ethics review board reviewed our study protocol (Jiangxi Provincial People’s Hospital: IRB 2021-066) and waived duplicate subject informed consent signing.

**Figure 1:**
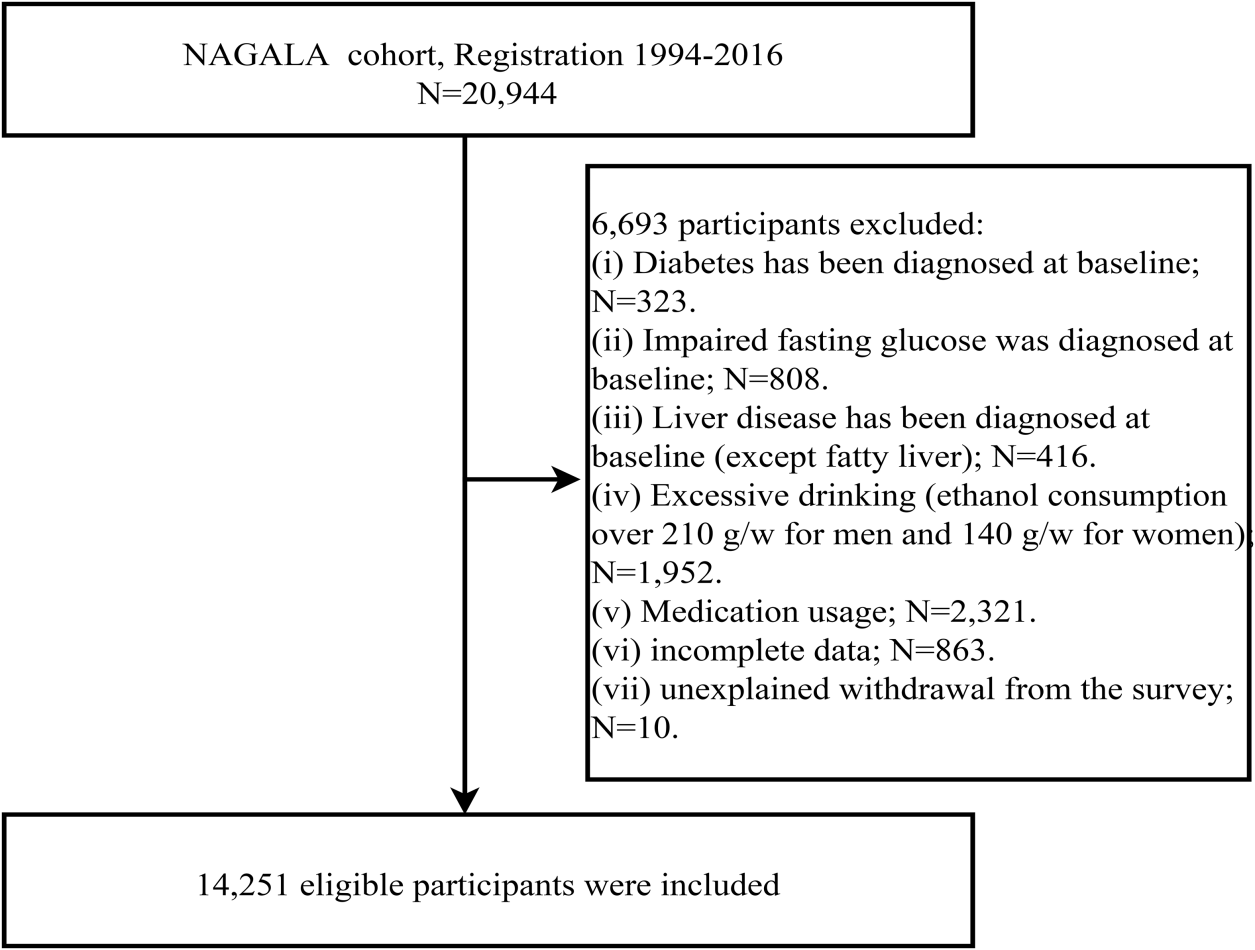
Flow chart for inclusion and exclusion of study participants.

We downloaded raw data from an online database, and the original cohort included a total of 20,944 participants spanning from 1994 to 2016. Based on new research hypotheses, we further developed new inclusion criteria on the previous study. Participants were excluded if they met the following baseline criteria: (1) excessive alcohol consumption (males: weekly alcohol intake ≥ 210g; females: weekly alcohol intake ≥ 140g) (n=1,952), (2) diagnosed diabetes or fasting plasma glucose (FPG) ≥ 6.1 mmol/L (n=1,131) (32), (3) viral or alcoholic hepatitis (n=416), (4) medication use (n=2,321), and (5) participants with missing data or reasons for withdrawal that were unclear (n=873). Ultimately, a total of 14,251 participants were included in this study. The flowchart illustrating the inclusion and exclusion process of the study population was shown in **Figure 1**.

### Data collection

The data collection methods have been described in detail in previous studies (31). In brief, standardized questionnaires were prepared by trained personnel to collect information on demographic characteristics, health conditions (medical history and medication use), and lifestyle habits (smoking status, drinking status, and exercise habits) from the participants. Information on general physical measurements (including height, weight, waist circumference (WC), blood pressure (BP), etc.) was obtained by trained professionals using standardized methods. Laboratory test data, including blood glucose parameters [FPG and hemoglobin A1c (HbA1c)], lipid parameters [high-density lipoprotein cholesterol (HDL-C), triglyceride (TG), and total cholesterol (TC)], and liver enzyme parameters (ALT, AST, and GGT), were measured using automated biochemical analyzers. Blood samples were collected by trained medical personnel after an overnight fast of 8 hours.

### Definition of lifestyle factors

Smoking status: Participants were categorized into three groups based on their smoking history at baseline: none, past, and current.

Exercise habits: Participants were classified as having exercise habits or no exercise habits based on their regular exercise frequency per week (33).

Drinking status: Participants’ alcohol intake per week in the previous month was divided into three categories: none/small (<40 g/week), moderate (40-140 g/week), and heavy (140-209 g/week) (34).

### Definition of NAFLD

In the current study, after excluding liver diseases caused by medications, excessive alcohol intake, and other known reasons, NAFLD was diagnosed using Doppler ultrasound (35, 36). Specifically, in the absence of other information about the patient, experienced gastroenterologists scored four known criteria on abdominal color ultrasound, including deep attenuation (0-2 points), hepatorenal echo contrast (0-4 points), liver brightness (0-4 points), and vascular blurring (0-2 points). A total score exceeding 2 points indicated a diagnosis of NAFLD (37).

### Statistical analysis

First, we compared the baseline differences between the NAFLD and non-NAFLD groups separately for males and females. Additionally, we also divided the participants into two groups according to whether they had NAFLD or not, and compared the differences in the baseline data between the two groups of males and females. Baseline data were presented as mean (standard deviation), median (interquartile range), and frequency (%), depending on the distribution of the variables. Inverse probability of treatment weighting was used for quantification and evaluation of between-group differences, calculating weighted standardized differences, with a significant difference defined as a standardized difference >10% (38, 39).

Logistic regression analysis was used to estimate the ORs and 95% CIs to assess the associations between liver enzymes (ALT, AST, and GGT) and NAFLD in the overall population and separately for males and females. The ORs per standard deviation increase in each liver enzyme were recorded. Collinearity screening was performed before constructing the multivariable logistic regression models, and covariates with multicollinearity were not included in the model adjustment, with multicollinearity defined as a variance inflation factor >5 (**Supplementary Tables 1–3**) (40). The multivariable-adjusted logistic regression models considered the influence of potential confounders, including demographic characteristics (age), lifestyle habits (smoking status, drinking status, and exercise habits), physical measurement parameters (height, weight, WC, BP), and laboratory biochemical indicators (blood glucose: FPG and HbA1c; blood lipids: HDL-C, TG, and TC). Additionally, we further conducted interaction tests to examine the gender differences in the NAFLD risk associated with ALT, AST, and GGT.

The discriminative performance of ALT, AST, and GGT for identifying NAFLD in males and females was evaluated using ROC curves, with corresponding AUC and 95% CI, thresholds, sensitivity, and specificity recorded. In addition, we explored multiple liver enzyme combination screening models in both genders. Multivariate logistic regression models were used to construct diagnostic models for different liver enzyme combinations, and then ROC curves were plotted using the joint probabilities derived from the logistic regression models (41). The Delong test was used to compare the significance of the AUCs between different models (42).

For data processing and statistical analysis, we used R version 4.2.1 and Empower(R) version 2.20. Statistical significance was defined as *P* < 0.05 (two-tailed).

## Results

### Baseline characteristics of the study population

Total of the 20,944 participants in the NAGALA database, we excluded 6,693 individuals and ultimately included 14,251 participants, including 6,840 females [478 (6.99%) with NAFLD] and 7,411 males [2,029 (27.38%) with NAFLD].

**Table 1** summarizes the baseline characteristics of males and females stratified by NAFLD. Consistent with the expected results, significant differences were observed in multiple covariates between the NAFLD and non-NAFLD groups in both males and females. Specifically, in female participants, the NAFLD group had a higher mean age and lower height compared to the non-NAFLD group. Other parameters, including weight, BMI, WC, and BP, were higher in the NAFLD group. In terms of lifestyle habits, the NAFLD group had a higher rate of drinking, while there were no significant differences between the two groups in terms of exercise habits and smoking status. Regarding laboratory biochemical indicators, the NAFLD group had higher levels of liver enzymes (ALT, AST, and GGT), blood glucose (FPG and HbA1c), and blood lipids (TG and TC), while the HDL-C level was lower. In male participants, there were no significant differences in age and height between the NAFLD and non-NAFLD groups. Furthermore, the NAFLD group had a lower rate of exercise habits compared to the non-NAFLD group, and the differences in other covariates were similar to those in the female population.

**Table 1:** Baseline demographic, lifestyle, and laboratory characteristics in participants classified by the presence of different gender and incidence of NAFLD.

	Female			Male		
	Non-NAFLD	NAFLD	Standardize Difference (95% CI), %	Non-NAFLD	NAFLD	Standardize Difference (95% CI), %
Participants, n	6362	478		5382	2029	
Age, years	42.89 (8.72)*	47.64 (8.29)*	56 (46-65)	43.71 (9.27)*	44.11 (8.20)*	5 (0-10)
Height, cm	158.37(5.38)*	157.04(5.28)*	25 (16-34)	170.89(6.04)*	170.62(5.94)*	5 (-1-10)
Weight, kg	51.86 (7.06)*	63.17 (9.97)*	131 (121-141)	64.65 (8.34)*	74.30 (10.56)*	101 (96-107)
BMI, kg/m^2^	20.67 (2.57)*	25.58 (3.57)*	158 (148-168)	22.12 (2.42)*	25.48 (3.02)*	123 (117-128)
WC, cm	70.80 (7.30)*	83.27 (8.86)*	154 (144-163)	77.99 (6.77)*	86.62 (7.37)*	122 (116-127)
ALT, IU/L	13.00(11.00-17.00)^#^	19.00(15.00-26.00)^#^	63 (54-73)	18.00(14.00-23.00)^#^	29.00 (22.00-41.00) ^#^	93 (87-98)
AST, IU/L	16.00(13.00-19.00)^#^	18.00(15.00-22.00)^#^	35 (26-44)	17.00 (14.00-21.00) ^#^	21.00(17.00-26.00) ^#^	54 (49-60)
GGT, IU/L	12.00 (9.00-14.00) ^#^	15.00 (12.00-20.00)^#^	51 (41-60)	17.00 (14.00-24.00) ^#^	24.00(18.00-35.00) ^#^	44 (38-49)
HDL-C, mmol/L	1.66 (0.38)*	1.38 (0.34)*	79 (70-89)	1.35 (0.35)*	1.14 (0.25)*	68 (63-74)
TC, mmol/L	5.05 (0.86)*	5.56 (0.92)*	57 (47-66)	5.06 (0.84)*	5.41 (0.85)*	42 (37-47)
TG, mmol/L	0.54 (0.40-0.77) ^#^	1.02 (0.73-1.38) ^#^	96 (87, 106)	0.80 (0.58-1.16) ^#^	1.32 (0.91-1.86) ^#^	75 (70-80)
FPG, mmol/L	4.96 (0.38)*	5.27 (0.40)*	79 (70-88)	5.25 (0.37)*	5.42 (0.35)*	48 (43-53)
HbA1c, %	5.17 (0.32)*	5.42 (0.33)*	78 (69-87)	5.13 (0.31)*	5.27 (0.33)*	45 (40-50)
SBP, mmHg	108.42 (13.77)*	120.71 (16.04)*	82 (73-92)	116.04 (13.16)*	124.04 (14.46)*	58 (53-63)
DBP, mmHg	67.00 (9.48)*	75.11 (10.22)*	82 (73-92)	72.88 (9.32)*	78.44 (10.08)*	57 (52-62)
Exercise habits	1011 (15.89%)	68 (14.23%)	5 (-5-14)	1082 (20.10%)	309 (15.23%)	13 (8-18)
Drinking status			16 (6-25)			25 (20-30)
Non/small	5986 (94.09%)	465 (97.28%)		3731 (69.32%)	1623 (79.99%)	
Moderate	376 (5.91%)	13 (2.72%)		1096 (20.36%)	273 (13.45%)	
Heavy	0(0%)	0(0%)		555 (10.31%)	133 (6.55%)	
Smoking status			4 (-5-14)			6 (1-11)
None	5609 (88.16%)	427 (89.33%)		1952 (36.27%)	758 (37.36%)	
Past	382 (6.00%)	24 (5.02%)		1538 (28.58%)	615 (30.31%)	
Current	371 (5.83%)	27 (5.65%)		1892 (35.15%)	656 (32.33%)	

Values were expressed as mean (standard deviation)^*^ or median (interquartile range)^#^ or n (%).

NAFLD, non-alcoholic fatty liver disease; BMI, body mass index; WC, waist circumference; ALT, alanine aminotransferase; AST, aspartate aminotransferase; GGT, gamma-glutamyl transferase; HDL-C, high-density lipoprotein cholesterol; TC, total cholesterol; TG, triglyceride; HbA1c, hemoglobin A1c; FPG, fasting plasma glucose; SBP, systolic blood pressure; DBP, diastolic blood pressure.

**Table 2** displays the comparison of baseline clinical characteristics between males and females in NAFLD and non-NAFLD groups, respectively. In the NAFLD group, all covariates except BMI and exercise habits were significantly different between the genders. Specifically, in males, height, weight, WC, systolic BP, diastolic BP, ALT, AST, GGT, TG, and FPG were higher compared to females. Additionally, the rate of smoking and drinking was also higher in males, while age, HbA1c, HDL-C, and TC were higher in females. In the non-NAFLD group, there were no significant differences in age and TC between males and females. Furthermore, males had a higher BMI and a higher rate of exercise habits, while the differences in other covariates were similar to those in the NAFLD group.

**Table 2:** Baseline demographic, lifestyle, and laboratory characteristics in participants classified by incidence of NAFLD and the presence of different gender.

	Non-NAFLD			NAFLD		
	Female	Male	Standardize Difference (95% CI), %	Female	Male	Standardize Difference (95% CI), %
Participants, n	6362	5382		478	2029	
Age, years	42.89 (8.72)*	43.71 (9.27)*	9 (5-13)	47.64 (8.29)*	44.11 (8.20)*	43 (33-53)
Height, cm	158.37 (5.38)*	170.89 (6.04)*	219 (214-223)	157.04 (5.28)*	170.62 (5.94)*	242 (230-254)
Weight, kg	51.86 (7.06)*	64.65 (8.34)*	166 (161-170)	63.17 (9.97)*	74.30 (10.56)*	108 (98-119)
BMI, kg/m^2^	20.67 (2.57)*	22.12(2.42)*	58 (54-62)	25.58 (3.57)*	25.48 (3.02)*	3 (-7-13)
WC, cm	70.80 (7.30)*	77.99 (6.77)*	102 (98-106)	83.27 (8.86)*	86.62 (7.37)*	41 (31-51)
ALT, IU/L	13.00(11.00-17.00)^#^	18.00(14.00-23.00)^#^	51 (47-55)	19.00 (15.00-26.00)^#^	29.00(22.00-41.00)^#^	73 (62-83)
AST (IU/L)	16.00(13.00-19.00)^#^	17.00(14.00-21.00)^#^	24 (20-27)	18.00 (15.00-22.00)^#^	21.00(17.00-26.00)^#^	30 (20-40)
GGT, IU/L	12.00(9.00-14.00)^#^	17.00(14.00-24.00)^#^	65 (61-69)	15.00 (12.00-20.00)^#^	24.00(18.00-35.00)^#^	63 (53-73)
HDL-C, mmol/L	1.66 (0.38)*	1.35 (0.35)*	87 (83-91)	1.38 (0.34)*	1.14 (0.25)*	81 (71-91)
TC, mmol/L	5.05 (0.86)*	5.06 (0.84)*	1 (-2-5)	5.56 (0.92)*	5.41 (0.85)*	16 (6-26)
TG, mmol/L	0.54(0.40-0.77)^#^	0.80(0.58-1.16)^#^	66 (62-69)	1.02 (0.73-1.38)^#^	1.32(0.91-1.86)^#^	44 (34-54)
FPG, mmol/L	4.96 (0.38)*	5.25 (0.37)*	78 (74-81)	5.27 (0.40)*	5.42 (0.35)*	40 (30-50)
HbA1c, %	5.17 (0.32)*	5.13 (0.31)*	12 (8-16)	5.42 (0.33)*	5.27 (0.33)*	45 (35-55)
SBP, mmHg	108.42 (13.77)*	116.04 (13.16)*	57 (53-60)	120.71 (16.04)*	124.04 (14.46)*	22 (12-32)
DBP, mmHg	67.00 (9.48)*	72.88 (9.32)*	63 (59-66)	75.11 (10.22)*	78.44 (10.08)*	33 (23-43)
Exercise habits	1011 (15.89%)	1082 (20.10%)	11 (7-15)	68 (14.23%)	309 (15.23%)	3(-7-13)
Drinking status			70 (67-74)			58 (48-68)
Non/small	5986 (94.09%)	3731 (69.32%)		465 (97.28%)	1623 (79.99%)	
Moderate	376 (5.91%)	1096 (20.36%)		13 (2.72%)	273 (13.45%)	
Heavy	0 (0.00%)	555 (10.31%)		0 (0.00%)	133 (6.55%)	
Smoking status			127 (123-131)			128 (118-139)
None	5609 (88.16%)	1952 (36.27%)		427 (89.33%)	758 (37.36%)	
Past	382 (6.00%)	1538 (28.58%)		24 (5.02%)	615 (30.31%)	
Current	371 (5.83%)	1892 (35.15%)		27 (5.65%)	656 (32.33%)	

Values were expressed as mean (standard deviation)* or median (interquartile range)^#^ or n (%).

all abbreviations as in **Table 1**.

### Association of ALT, AST, and GGT with NAFLD in male and female participants

Multivariable logistic regression analysis was performed to examine the associations between liver enzymes and NAFLD by gender (**Table 3**). In the logistic regression analysis adjusted for demographic characteristics (age), lifestyle habits (smoking status, drinking status, and exercise habits), physical measurement parameters (height, BMI, systolic BP), and laboratory biochemical indicators (blood glucose: FPG and HbA1c; blood lipids: HDL-C, TG, and TC), ALT, AST, and GGT were positively associated with NAFLD in both male and female populations. In female participants, ALT showed the most significant association with NAFLD (OR=1.60, 95% CI: 1.35-1.89), followed by GGT (OR=1.33, 95% CI: 1.15-1.52) and AST (OR=1.09, 95% CI: 1.00-1.18). In male participants, ALT exhibited the strongest association with NAFLD (OR=2.19, 95% CI: 2.01-2.39), followed by AST (OR=1.38, 95% CI: 1.28-1.49) and GGT (OR=1.14, 95% CI: 1.08-1.20). Furthermore, interaction analysis showed significant differences in the association of ALT, AST, and GGT with NAFLD risk between males and females (*P* for interaction <0.05), with ALT and AST showing a significant association with NAFLD in males, while GGT showed a significant association with NAFLD in females.

**Table 3:** Logistic regression analyses for the association between ALT, AST and GGT with incident NAFLD grouped by gender.

	Odds ratios (95% confidence interval)		*P-*interaction
	Female	Male	
ALT (Per SD increase)	1.60 (1.35-1.89)	2.19 (2.01-2.39)	0.01
AST (Per SD increase)	1.09 (1.00-1.18)	1.38 (1.28-1.49)	<0.01
GGT (Per SD increase)	1.33 (1.15-1.52)	1.14 (1.08-1.20)	0.04

Abbreviations as in **Table 1**.

Adjusted for age, height, BMI, exercise habits, drinking status, smoking status, HDL-C, TC, TG, FPG, HBA1C and SBP.

### ROC analysis of ALT, AST, and GGT in identifying NAFLD

**Figure 2:**
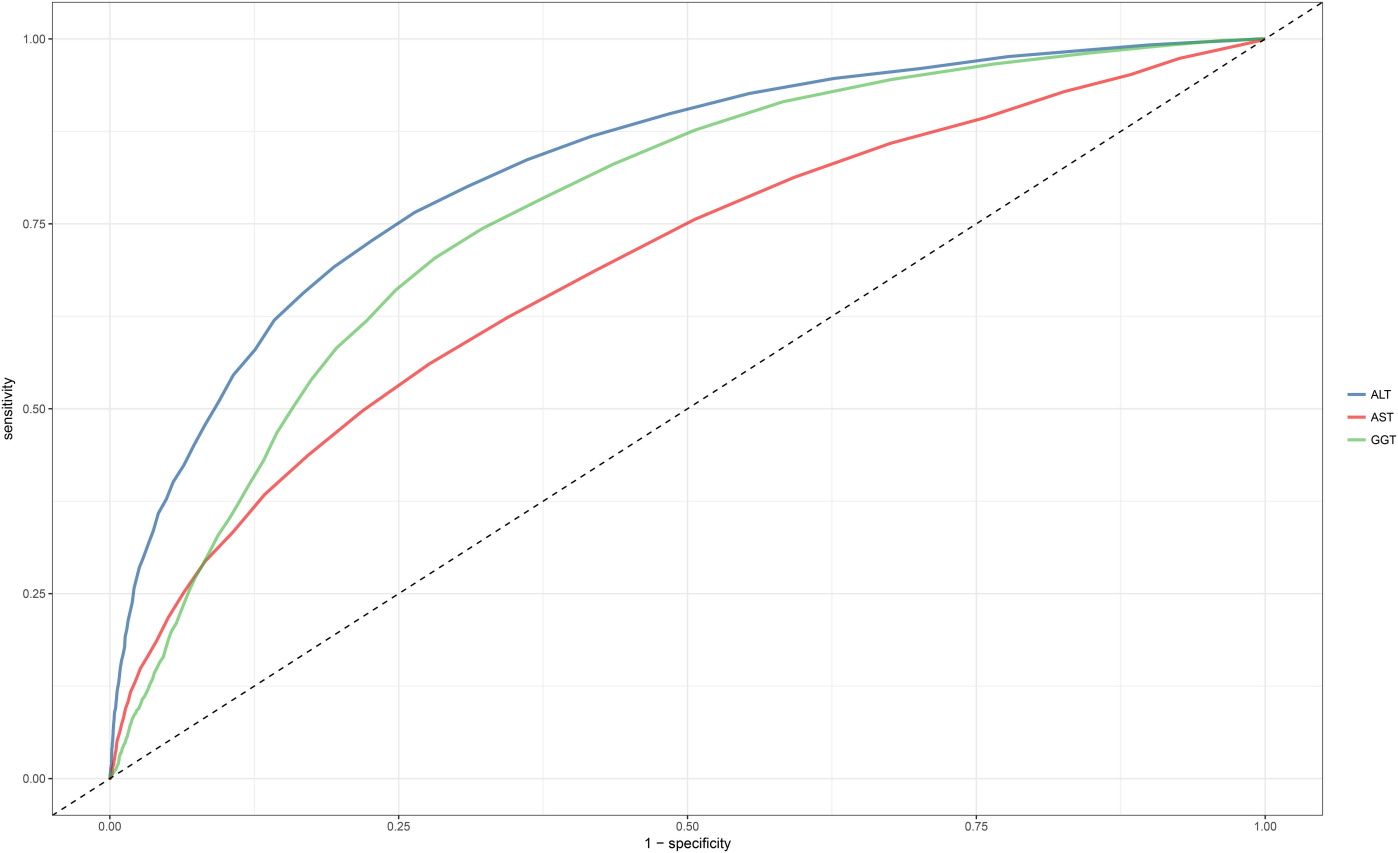
Area under the receiver operating characteristic curve for ALT, AST, and GGT for identification of NAFLD in the entire population. ALT, alanine aminotransferase; AST, aspartate aminotransferase; GGT, gamma-glutamyl transferase; NAFLD, Nonalcoholic fatty liver disease.

**Figure 3:**
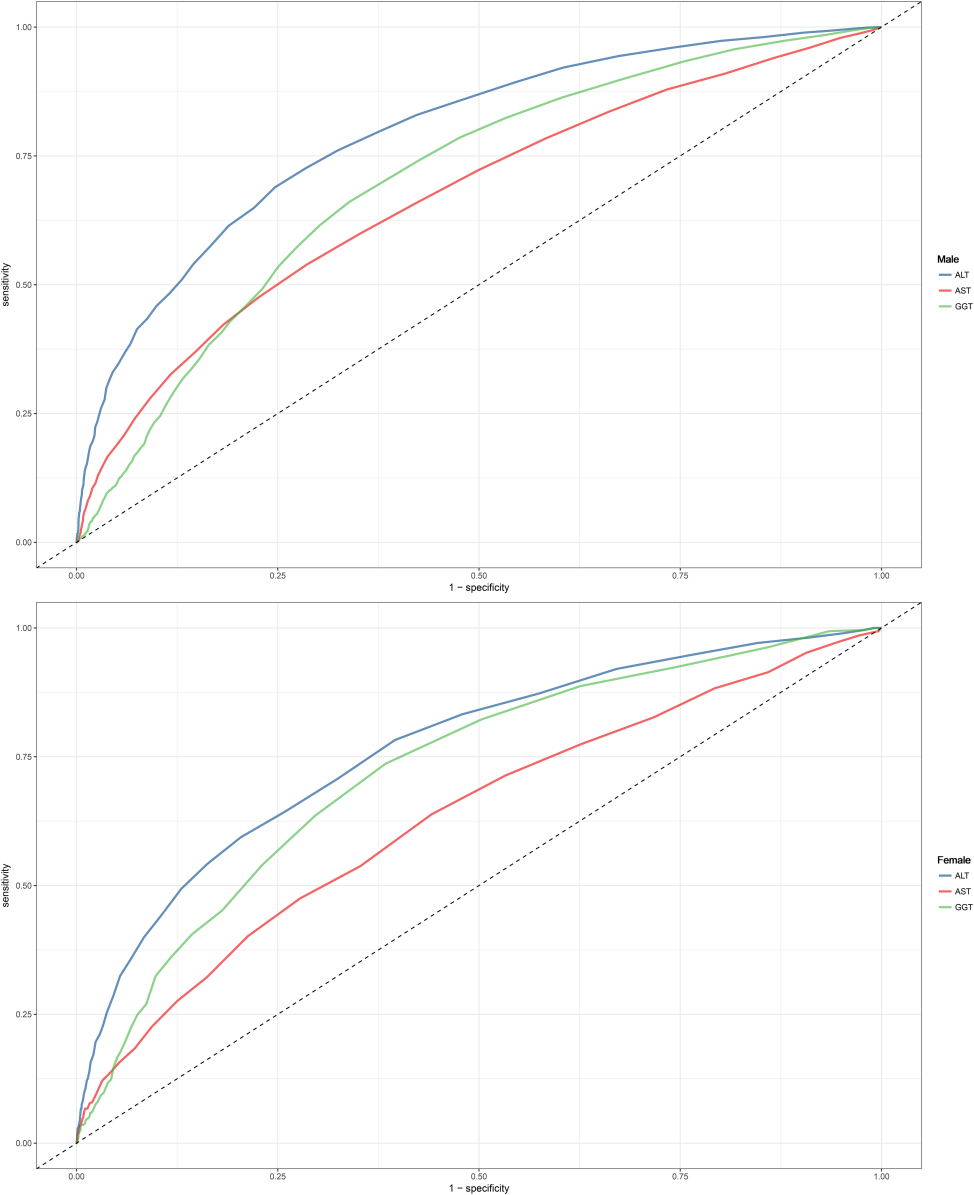
Area under the receiver operating characteristic curve for ALT, AST, and GGT for identification of NAFLD in the male and female. ALT, alanine aminotransferase; AST, aspartate aminotransferase; GGT, gamma-glutamyl transferase; NAFLD, Nonalcoholic fatty liver disease.

ROC analysis was conducted for the three liver enzymes to determine their ability to detect NAFLD, and comparisons were made using the Delong test. **Table 4** presents the AUC, 95% CI, thresholds, sensitivity, and specificity of the liver enzyme parameters for identifying NAFLD in the overall population (**Figure 2**) and in males and females (**Figure 3**). Overall, ALT had the highest AUC value in the overall population and in both males and females, with AUCs of 0.83 (95% CI: 0.82-0.84) in the overall population, 0.77 (95% CI: 0.74-0.79) in females, and 0.79 (95% CI: 0.78-0.80) in males. The corresponding optimal cutoff values were 19.5 U/L, 17.5 U/L, and 23.5 U/L, with specificities of 0.74, 0.80, and 0.75, and sensitivities of 0.77, 0.59, and 0.69, respectively. Further analysis revealed that the AUC values of the liver enzymes in different populations followed the order of ALT > GGT > AST from highest to lowest. Additionally, the Delong test showed that, in the overall population and in both males and females, ALT had significantly better performance in identifying NAFLD compared to AST and GGT when used as a single parameter (all Delong *P* < 0.05).

**Table 4:** Areas under the receiver operating characteristic curves, 95%CI, best threshold, sensitivity and specificity for ALT, AST and GGT identifies NAFLD risk for all participants, females and males.

	AUC	95%CI	Best threshold	Specificity	Sensitivity
All participants (N=14,251)					
ALT	0.83	(0.82-0.84)	19.50	0.74	0.77
AST	0.69*	(0.68-0.70)	19.50	0.72	0.56
GGT	0.77*	(0.76-0.78)	17.50	0.72	0.70
Female (N=6,840)					
ALT	0.77	(0.74-0.79)	17.50	0.80	0.59
AST	0.63*	(0.61-0.66)	18.50	0.72	0.47
GGT	0.72*	(0.70-0.75)	12.50	0.61	0.74
Male (N=7,411)					
ALT	0.79	(0.78-0.80)	23.50	0.75	0.69
AST	0.67*	(0.66-0.68)	20.50	0.71	0.54
GGT	0.70*	(0.69-0.72)	20.50	0.66	0.66

AUC, area under the receiver operating characteristic curve; CI, confidence interval; other abbreviations as in **Table 1**.

*P<0.05, compare with ALT.

### ROC analysis of different combinations of liver enzymes in identifying NAFLD in males and females

Regression equations for the combinations of liver enzymes were obtained through multivariate logistic regression. In the previous multivariate regression analysis, ALT, AST, and GGT were found to be independently associated with NAFLD. To explore the most suitable combination of liver enzymes for NAFLD screening in males and females, several combination models were constructed, and logistic regression models were used to calculate the joint probabilities. **Table 5** presents the AUC, 95% CI, sensitivity, and specificity of different combinations of liver enzymes in identifying NAFLD in males and females. The results showed that, compared to using ALT alone for identifying NAFLD, only the combination of ALT and GGT improved the identification performance (Delong *P*-value < 0.05). It is worth noting that although there were statistical differences, the improvement in AUC was not substantial [males: ALT (0.79) vs ALT+GGT (0.79); females: ALT (0.77) vs ALT+GGT (0.77)].

**Table 5:** Areas under the receiver operating characteristic curves, 95%CI, sensitivity and specificity for different combination of liver function enzymes identifies NAFLD risk for females and males.

	AUC	95%CI	Specificity	Sensitivity	Delong *P*-value
Female (N=6,840)					
ALT	0.77	(0.74-0.79)	0.80	0.59	
ALT+AST	0.76	(0.73-0.78)	0.80	0.61	0.33
ALT+GGT	0.77	(0.74-0.79)	0.65	0.75	<0.01
AST+ALT+GGT	0.76	(0.74-0.79)	0.83	0.59	0.55
Male (N=7,411)					
ALT	0.79	(0.78-0.80)	0.75	0.69	
ALT+AST	0.80	(0.79-0.81)	0.67	0.78	0.17
ALT+GGT	0.79	(0.78-0.81)	0.72	0.73	0.04
AST+ALT+GGT	0.80	(0.69-0.81)	0.69	0.76	0.15

AUC, area under the receiver operating characteristic curve; CI, confidence interval; other abbreviations as in **Table 1**.

## Discussion

In this large-scale epidemiological study based on data from various anthropometric and biochemical measurements taken during health examinations of individuals at the Murakami Memorial Hospital of Gifu, after adjusting for several confounding factors such as demographic characteristics, lifestyle, physical measurements, and laboratory biochemical indicators, higher liver enzymes (including ALT, AST, and GGT) were independently associated with both male and female NAFLD. In the male population, the association between liver enzymes and NAFLD was from strong to weak: ALT > AST > GGT, while in the female population it was ALT > GGT > AST in that order; further interactive tests showed that ALT was more suitable for assessing NAFLD risk in males than in females. Furthermore, ROC analysis showed that ALT had the highest accuracy in identifying NAFLD, especially in males. After further combining the liver enzyme parameters, we found that the combination of ALT and GGT had better recognition performance for NAFLD in both genders compared to ALT alone (all Delong *P*-values <0.05). However, it should be noted that although there were statistical differences, the improvement in AUC values was not great.

Liver enzymes are well-known biochemical indicators positively correlated to NAFLD (20, 21, 43–48), and previous studies and guidelines have recommended using liver enzymes as preliminary screening parameters for NAFLD (13, 49). However, there are significant differences in the levels and activities of different liver enzymes between male and female populations. Therefore, quantifying the association between liver enzyme parameters and NAFLD in both genders and exploring the most suitable liver enzyme markers for screening NAFLD in both genders are of practical significance for precise medical treatment of NAFLD. However, currently, there is limited comparative research on the strength of the association between liver enzymes and NAFLD, especially based on gender stratification. In a cross-sectional study of 210 diabetic patients in Nepal (50), Mandal et al. found that ALT was independently associated with NAFLD, while AST and GGT showed no significant association. Furthermore, in another cross-sectional study of obese children in Italy (51), the results also indicated that only ALT was independently associated with NAFLD. After further gender stratification, we found that only one cross-sectional study involving employees of Tongji University in China (52) evaluated gender differences. In this study, Bi et al. evaluated the association between ALT, AST, GGT, and NAFLD in both genders and found that GGT had a stronger association with NAFLD than ALT and AST in both genders. It is worth noting that the study populations in the three studies mentioned above were highly heterogeneous, and there is still lack of evidence in the general population. In the current cross-sectional study, using health examination data from the general population, we obtained results consistent with the majority of previous studies, namely, ALT, AST, and GGT were independently associated with NAFLD in both genders. Additionally, we found that ALT was more significant associated with NAFLD than AST and GGT in both males and females. These findings collectively emphasized a key point: in the general population, ALT may be the most sensitive liver enzyme indicator for evaluating NAFLD.

Some previous studies have reported on the diagnostic/identification performance of ALT, AST, and GGT for NAFLD (48–51). However, only one cross-sectional study analyzed and compared the diagnostic performance of ALT, AST, and GGT for NAFLD in both genders. In this study, Chien-Min et al. first analyzed the risk factors associated with NAFLD and then further evaluated the diagnostic value of various indicators, including ALT, AST, and GGT, for NAFLD using ROC curves. The results showed that ALT had higher accuracy in identifying NAFLD than AST and GGT in the overall population. However, when further stratifying by gender, different results were found. In the male population, GGT had higher accuracy in identifying NAFLD, followed by ALT and AST; while in the female population, ALT had higher accuracy, followed by AST, and GGT had the lowest accuracy (49). In contrast to the previous study’s findings, in the current cross-sectional study, we found that ALT had higher accuracy than AST and GGT in identifying NAFLD in both males and females. The differences in research findings may be due to different study designs and population heterogeneity. Additionally, Chien-Min et al.’s study only included 236 healthy participants (49), while in the current study, we included a total of 14,521 participants, which provided sufficient sample size to enhance statistical power and improved the accuracy of the results. Furthermore, a larger sample size can better validate the reliability of our findings.

There have been no previous studies reporting on the analysis of the identification performance of different combinations of liver enzymes for NAFLD in both genders. In the current study, we found that using the ALT+GGT combination in both genders significantly improved the identification accuracy of NAFLD compared to using ALT alone. However, this improvement was not substantial in numerical values and required complex calculations. Additionally, using ALT alone to identify NAFLD in both genders resulted in an AUC above 0.76, especially in males where the AUC approached 0.8. This indicated that using ALT alone as a preliminary screening tool for high-risk populations of NAFLD in clinical settings already has high accuracy. Although the ALT+GGT combination improved the accuracy of NAFLD screening, it required complex formula calculations and evaluations, which increased the workload for primary care physicians and reduced time efficiency. Furthermore, complex calculations tended to lead to brain and body fatigue in primary care physicians, which may limit the usefulness of liver enzyme combinations in clinical settings.

Our results provided valuable information for the primary care-based screening strategy for NAFLD in both genders. First, the results of logistic regression analysis and ROC analysis collectively emphasized that ALT may be the most sensitive liver enzyme indicator for NAFLD in both genders. Additionally, using ALT alone as a preliminary screening tool avoids complex calculations, ensuring high accuracy while maintaining screening efficiency. For identifying NAFLD, we recommend an ALT threshold of 23.5 for males and 17.5 for females, which were lower than the previously recommended upper limits of serum ALT levels for Asian populations (53–55). In recent years, with the rapid increase in the prevalence of NAFLD, there has been more discussion on lowering the upper limit of serum ALT levels (56–58). However, some concerns have been raised about that lowering the upper limit of ALT will increase the proportion of people at risk for NAFLD and increase unnecessary medical evaluation and costs (56), similar to the opposing opinions on lowering the thresholds for blood glucose (59) and BP (60) in recent years. It should be noted that although lowering the upper limit of ALT for health may increase the inclusion of non-NAFLD patients, having a normal ALT level does not guarantee the absence of NAFLD. Several studies have shown that a considerable proportion of patients with fatty liver have ALT levels below the laboratory-set upper limit, especially in some NAFLD patients with advanced histological damage (53, 55, 61), who require more attention. According to the stepwise screening method recommended by the World Gastroenterology Organization (13), when the biochemical ALT exceeds the recommended threshold, there should be a high suspicion of the possibility of NAFLD, and primary care physicians should pay sufficient attention and guide patients to further examinations such as liver ultrasound or liver biopsy, reducing unnecessary tests. In conclusion, our study confirmed the effectiveness of ALT in identifying NAFLD in both genders. According to the results of the current study, we recommend using ALT as an initial screening tool for NALFD in both genders (especially in males) and conducting further strict screening of suspicious individuals exceeding the threshold, which can reduce unnecessary examinations, avoid the waste of medical resources, and reduce national economic and medical expenditure in NAFLD prevention and screening, especially for some countries and regions with underdeveloped medical conditions.

The circadian rhythm variations in liver enzymes should be given attention, as they may influence the gender disparity in NAFLD prevalence. It is well known that liver enzymes exhibit significant circadian rhythmicity (with maximum diurnal variation exceeding 10%) (62), and such fluctuations might be linked to the expression levels of liver genes and the regulation of gonadal steroid activation and tissue effects (63–65). Furthermore, reliable evidence suggests significant gender differences in circadian rhythms (65, 66), with sex hormones potentially regulating circadian parameters in a gender-dependent manner. Classical studies on circadian rhythms have also demonstrated the gender-differential roles of gonadal steroid activation and tissue effects regulation in daily rhythms (67–69), and these differences might relate to the overall gender-dimorphic gene expression rhythms. A study on the 24-hour gene expression rhythms in humans revealed that females exhibit higher transcriptional rhythmicity than males, especially in the liver (70). The detailed mechanisms of circadian rhythms’ impact on NAFLD remain unclear. In fact, the regulation of circadian rhythms is multifaceted and complex, encompassing various lifestyle factors such as smoking, alcohol consumption, and staying up late, all of which can disrupt the body’s circadian rhythms (71, 72); this constitutes a complex network of relationships, requiring further research to elucidate the role of circadian rhythms in the development of NAFLD. Fortunately, recent research on circadian rhythms has been very active, and new findings are vitally important for the precise screening of NAFLD and enhancing public awareness for its prevention. Considering our study’s liver enzyme levels were measured after an overnight fast of 8 hours, our previous recommendations for liver enzyme thresholds should be given attention in future research.

### Advantages and limitations

This study has the following advantages: Firstly, we used a large sample of health examination data from the general population, which has good representativeness and applicability. Secondly, we conducted gender-stratified analyses, revealing the differences in the strength of association between liver enzymes and NAFLD in different gender populations, and exploring the optimal liver enzyme combination model for identifying NAFLD in both genders.

However, we also acknowledge some limitations of this study. Firstly, the thresholds for liver enzymes have racial and population differences, and further evaluation of the optimal screening thresholds for the two genders need to be conducted in other populations. Secondly, due to the lack of other liver function markers such as alkaline phosphatase and lactate dehydrogenase, we could not compare their association strengths with NAFLD. Additionally, in this study, we used ultrasound examination for diagnosing NAFLD; although ultrasound is considered a reliable and accurate test for moderate to severe fatty liver (73), it may miss some participants with mild fatty liver, especially when hepatic steatosis is less than 20% (74). Finally, although statistical results showed that using the ALT+GGT combination significantly improved the identification accuracy of NAFLD, the clinical significance of this change has not been determined, and further research is needed to evaluate its clinical benefits.

## Conclusion

Our study revealed significant gender differences in the associations of the three commonly used liver enzyme markers with NAFLD. In both genders, the use of ALT alone may be the simplest and most effective tool for screening NAFLD, especially in males.

## Data availability statement

The datasets presented in this study can be found in online repositories. The names of the repository/repositories and accession number(s) can be found in the article/**Supplementary Material**.

## Ethics statement

The studies involving humans were approved by the Ethics Committee of Jiangxi Provincial People’s Hospital. The studies were conducted in accordance with the local legislation and institutional requirements. Since the current data set has been anonymized, the Institutional Ethics Committee of Jiangxi Provincial People’s Hospital exempted subjects from repeatedly signing the informed consent application in accordance with local laws and regulations.

## Author contributions

JQ: Data curation, Formal Analysis, Software, Validation, Visualization, Writing – original draft. MK: Formal Analysis, Validation, Visualization, Writing – review & editing. SH: Validation, Writing – review & editing. CY: Validation, Writing – review & editing. CW: Formal Analysis, Validation, Visualization, Writing – review & editing. XH: Formal Analysis, Validation, Visualization, Writing – review & editing. GS: Conceptualization, Project administration, Supervision, Writing – original draft. YZ: Conceptualization, Formal Analysis, Methodology, Project administration, Supervision, Visualization, Writing – review & editing.

## References

[1] Finck BN. Targeting metabolism, insulin resistance, and diabetes to treat nonalcoholic steatohepatitis. Diabetes (2018) 67(12):2485–93. doi: 10.2337/dbi18-0024 PMC6245219 30459251

[2] Le MH Le DM Baez TC Wu Y Ito T Lee EY. Global incidence of non-alcoholic fatty liver disease: a systematic review and meta-analysis of 63 studies and 1,201,807 persons. J Hepatol (2023) 79(2):287–95. doi: 10.1016/j.jhep.2023.03.040 37040843

[3] Powell EE Wong VW Rinella M. Non-alcoholic fatty liver disease. Lancet (2021) 397(10290):2212–24. doi: 10.1016/S0140-6736(20)32511-3 33894145

[4] Maurice J Manousou P. Non-alcoholic fatty liver disease. Clin Med (Lond) (2018) 18(3):245–50. doi: 10.7861/clinmedicine.18-3-245 PMC6334080 29858436

[5] Rinella ME. Nonalcoholic fatty liver disease: a systematic review. JAMA (2015) 313(22):2263–73. doi: 10.1001/jama.2015.5370 26057287

[6] Lonardo A Nascimbeni F Ballestri S Fairweather D Win S Than TA. Sex differences in nonalcoholic fatty liver disease: state of the art and identification of research gaps. Hepatology (2019) 70(4):1457–69. doi: 10.1002/hep.30626 PMC6766425 30924946

[7] Burra P Bizzaro D Gonta A Shalaby S Gambato M Morelli MC. Clinical impact of sexual dimorphism in non-alcoholic fatty liver disease (NAFLD) and non-alcoholic steatohepatitis (NASH). Liver Int (2021) 41(8):1713–33. doi: 10.1111/liv.14943 33982400

[8] Lefebvre P Staels B. Hepatic sexual dimorphism - implications for non-alcoholic fatty liver disease. Nat Rev Endocrinol (2021) 17(11):662–70. doi: 10.1038/s41574-021-00538-6 34417588

[9] Carr RM Oranu A Khungar V. Nonalcoholic fatty liver disease: pathophysiology and management. Gastroenterol Clin North Am (2016) 45(4):639–52. doi: 10.1016/j.gtc.2016.07.003 PMC5127277 27837778

[10] Ratziu V Cadranel JF Serfaty L Denis J Renou C Delassalle P. A survey of patterns of practice and perception of NAFLD in a large sample of practicing gastroenterologists in France. J Hepatol (2012) 57(2):376–83. doi: 10.1016/j.jhep.2012.03.019 22521354

[11] Sanyal D Mukherjee P Raychaudhuri M Ghosh S Mukherjee S Chowdhury S. Profile of liver enzymes in non-alcoholic fatty liver disease in patients with impaired glucose tolerance and newly detected untreated type 2 diabetes. Indian J Endocrinol Metab (2015) 19(5):597–601. doi: 10.4103/2230-8210.163172 26425466 PMC4566337

[12] Kalinich M Bhan I Kwan TT Miyamoto DT Javaid S LiCausi JA. An RNA-based signature enables high specificity detection of circulating tumor cells in hepatocellular carcinoma. Proc Natl Acad Sci U.S.A. (2017) 114(5):1123–8. doi: 10.1073/pnas.1617032114 PMC5293050 28096363

[13] Review Team LaBrecque DR Abbas Z Anania F Ferenci P Khan AG. World Gastroenterology Organisation global guidelines: Nonalcoholic fatty liver disease and nonalcoholic steatohepatitis. J Clin Gastroenterol (2014) 48(6):467–73. doi: 10.1097/MCG.0000000000000116 24921212

[14] Westfall E Jeske R Bader AR. Nonalcoholic fatty liver disease: common questions and answers on diagnosis and management. Am Fam Physician (2020) 102(10):603–12. 33179890

[15] Kew MC. Serum aminotransferase concentration as evidence of hepatocellular damage. Lancet (2000) 355(9204):591–2. doi: 10.1016/S0140-6736(99)00219-6 10696975

[16] Hadizadeh F Faghihimani E Adibi P. Nonalcoholic fatty liver disease: Diagnostic biomarkers. World J Gastrointest Pathophysiol (2017) 8(2):11–26. doi: 10.4291/wjgp.v8.i2.11 28573064 PMC5437499

[17] Manne V Handa P Kowdley KV. Pathophysiology of nonalcoholic fatty liver disease/nonalcoholic steatohepatitis. Clin Liver Dis (2018) 22(1):23–37. doi: 10.1016/j.cld.2017.08.007 29128059

[18] Leoni S Tovoli F Napoli L Serio I Ferri S Bolondi L. Current guidelines for the management of non-alcoholic fatty liver disease: A systematic review with comparative analysis. World J Gastroenterol (2018) 24(30):3361–73. doi: 10.3748/wjg.v24.i30.3361 PMC6092580 30122876

[19] Athyros VG Giouleme O Ganotakis ES Elisaf M Tziomalos K Vassiliadis T. Safety and impact on cardiovascular events of long-term multifactorial treatment in patients with metabolic syndrome and abnormal liver function tests: a *post hoc* analysis of the randomised ATTEMPT study. Arch Med Sci (2011) 7(5):796–805. doi: 10.5114/aoms.2011.25554 22291824 PMC3258797

[20] Lazo M Rubin J Clark JM Coresh J Schneider AL Ndumele C. The association of liver enzymes with biomarkers of subclinical myocardial damage and structural heart disease. J Hepatol (2015) 62(4):841–7. doi: 10.1016/j.jhep.2014.11.024 PMC4373587 25433159

[21] Lazo M Clark JM. The epidemiology of nonalcoholic fatty liver disease: a global perspective. Semin Liver Dis (2008) 28(4):339–50. doi: 10.1055/s-0028-1091978 18956290

[22] Dufour DR Lott JA Nolte FS Gretch DR Koff RS Seeff LB. Diagnosis and monitoring of hepatic injury. I. Performance characteristics of laboratory tests. Clin Chem (2000) 46(12):2027–49. doi: 10.1016/S0009-8981(00)00329-6 11106349

[23] Jones H Sprung VS Pugh CJ Daousi C Irwin A Aziz N. Polycystic ovary syndrome with hyperandrogenism is characterized by an increased risk of hepatic steatosis compared to nonhyperandrogenic PCOS phenotypes and healthy controls, independent of obesity and insulin resistance. J Clin Endocrinol Metab (2012) 97(10):3709–16. doi: 10.1210/jc.2012-1382 22837189

[24] Schwingel PA Zoppi CC Cotrim HP. Increased liver steatosis in anabolic-androgenic steroid users: more evidence towards toxicant-associated fatty liver disease development. Liver Int (2011) 31(8):1240–1. doi: 10.1111/j.1478-3231.2011.02552.x 21745295

[25] Kur P Kolasa-Wołosiuk A Misiakiewicz-Has K Wiszniewska B. Sex hormone-dependent physiology and diseases of liver. Int J Environ Res Public Health (2020) 17(8):2620. doi: 10.3390/ijerph17082620 32290381 PMC7216036

[26] Morán-Costoya A Proenza AM Gianotti M Lladó I Valle A. Sex differences in nonalcoholic fatty liver disease: estrogen influence on the liver-adipose tissue crosstalk. Antioxid Redox Signal (2021) 35(9):753–74. doi: 10.1089/ars.2021.0044 33736456

[27] McKenzie J Fisher BM Jaap AJ Stanley A Paterson K Sattar N. Effects of HRT on liver enzyme levels in women with type 2 diabetes: a randomized placebo-controlled trial. Clin Endocrinol (Oxf) (2006) 65(1):40–4. doi: 10.1111/j.1365-2265.2006.02543.x 16817817

[28] Kwo PY Cohen SM Lim JK. ACG clinical guideline: evaluation of abnormal liver chemistries. Am J Gastroenterol (2017) 112(1):18–35. doi: 10.1038/ajg.2016.517 27995906

[29] Oh RC Hustead TR Ali SM Pantsari MW. Mildly elevated liver transaminase levels: causes and evaluation. Am Fam Physician (2017) 96(11):709–15. 29431403

[30] Okamura T Hashimoto Y Hamaguchi M Obora A Kojima T Fukui M. Data from: Ectopic fat obesity presents the greatest risk for incident type 2 diabetes: a population-based longitudinal study. [Dataset]. Dryad (2019). doi: 10.5061/dryad.8q0p192 29717276

[31] Okamura T Hashimoto Y Hamaguchi M Obora A Kojima T Fukui M. Ectopic fat obesity presents the greatest risk for incident type 2 diabetes: a population-based longitudinal study. Int J Obes (Lond) (2019) 43(1):139–48. doi: 10.1038/s41366-018-0076-3 29717276

[32] American Diabetes Association. Standards of medical care in diabetes–2011. Diabetes Care (2011) 34 Suppl 1(Suppl 1):S11–61. doi: 10.2337/dc11-S011 PMC3006050 21193625

[33] Ryu S Chang Y Kim DI Kim WS Suh BS. gamma-Glutamyltransferase as a predictor of chronic kidney disease in nonhypertensive and nondiabetic Korean men. Clin Chem (2007) 53(1):71–7. doi: 10.1373/clinchem.2006.078980 17110470

[34] Hashimoto Y Hamaguchi M Kojima T Ohshima Y Ohbora A Kato T. Modest alcohol consumption reduces the incidence of fatty liver in men: a population-based large-scale cohort study. J Gastroenterol Hepatol (2015) 30(3):546–52. doi: 10.1111/jgh.12786 25238605

[35] Bedogni G Miglioli L Masutti F Tiribelli C Marchesini G Bellentani S. Prevalence of and risk factors for nonalcoholic fatty liver disease: the Dionysos nutrition and liver study. Hepatology (2005) 42(1):44–52. doi: 10.1002/hep.20734 15895401

[36] Chalasani N Younossi Z Lavine JE Diehl AM Brunt EM Cusi K. The diagnosis and management of non-alcoholic fatty liver disease: practice Guideline by the American Association for the Study of Liver Diseases, American College of Gastroenterology, and the American Gastroenterological Association. Hepatology (2012) 55(6):2005–23. doi: 10.1002/hep.25762 22488764

[37] Byrne CD. Ectopic fat, insulin resistance and non-alcoholic fatty liver disease. Proc Nutr Soc (2013) 72(4):412–9. doi: 10.1017/S0029665113001249 23668723

[38] Sato T Matsuyama Y. Marginal structural models as a tool for standardization. Epidemiology (2003) 14(6):680–6. doi: 10.1097/01.EDE.0000081989.82616.7d 14569183

[39] Muanda FT Weir MA Bathini L Blake PG Chauvin K Dixon SN. Association of baclofen with encephalopathy in patients with chronic kidney disease. JAMA (2019) 322(20):1987–95. doi: 10.1001/jama.2019.17725 PMC6865230 31705755

[40] Kim JH. Multicollinearity and misleading statistical results. Korean J Anesthesiol (2019) 72(6):558–69. doi: 10.4097/kja.19087 PMC6900425 31304696

[41] Chen J Pan Y Li G Xu W Zhang L Yuan S. Distinguishing between COVID-19 and influenza during the early stages by measurement of peripheral blood parameters. J Med Virol (2021) 93(2):1029–37. doi: 10.1002/jmv.26384 PMC7436548 32749709

[42] DeLong ER DeLong DM Clarke-Pearson DL. Comparing the areas under two or more correlated receiver operating characteristic curves: a nonparametric approach. Biometrics (1988) 44(3):837–45. doi: 10.2307/2531595 3203132

[43] Mansour-Ghanaei R Mansour-Ghanaei F Naghipour M Joukar F. Biochemical markers and lipid profile in nonalcoholic fatty liver disease patients in the PERSIAN Guilan cohort study (PGCS), Iran. J Family Med Prim Care (2019) 8(3):923–8. doi: 10.4103/jfmpc.jfmpc_243_18 PMC6482810 31041226

[44] Unalp-Arida A Ruhl CE. Noninvasive fatty liver markers predict liver disease mortality in the U.S. population. Hepatology (2016) 63(4):1170–83. doi: 10.1002/hep.28390 PMC4805455 26663021

[45] Zheng RD Lu LG Meng JR Huang JD Rao RC Xu CR. A clinical and pathological study of nonalcoholic fatty liver disease. Zhonghua Gan Zang Bing Za Zhi (2006) 14(6):449–52. 16792871

[46] Cruz MA Cruz JF Macena LB de Santana DS Oliveira CC Lima SO. Association of the nonalcoholic hepatic steatosis and its degrees with the values of liver enzymes and homeostasis model assessment-insulin resistance index. Gastroenterol Res (2015) 8(5):260–4. doi: 10.14740/gr685w PMC5051044 27785306

[47] Salman AA Aboelfadl SA Heagzy MA. New era for usage of serum liver enzymes as A promising horizon for the prediction of non-alcoholic fatty liver disease. Open Access Maced J Med Sci (2016) 4(3):348–52. doi: 10.3889/oamjms.2016.092 PMC5042614 27703554

[48] Chien-Min K Cheng-Chuan L. Clinical criteria correlated with the incidence of patients with non-alcoholic fatty liver disease. Ann Clin Lab Sci (2017) 47(2):191–200. 28442522

[49] European Association for the Study of the Liver (EASL) European Association for the Study of Diabetes (EASD) European Association for the Study of Obesity (EASO). EASL-EASD-EASO Clinical Practice Guidelines for the management of non-alcoholic fatty liver disease. J Hepatol (2016) 64(6):1388–402. doi: 10.1016/j.jhep.2015.11.004 27062661

[50] Mandal A Bhattarai B Kafle P Khalid M Jonnadula SK Lamicchane J. Elevated liver enzymes in patients with type 2 diabetes mellitus and non-alcoholic fatty liver disease. Cureus (2018) 10(11):e3626. doi: 10.7759/cureus.3626 30697502 PMC6347442

[51] Sartorio A Del Col A Agosti F Mazzilli G Bellentani S Tiribelli C. Predictors of non-alcoholic fatty liver disease in obese children. Eur J Clin Nutr (2007) 61(7):877–83. doi: 10.1038/sj.ejcn.1602588 17151586

[52] Bi WR Yang CQ Shi Q Xu Y Cao CP Ling J. Large-scale analysis of factors influencing nonalcoholic fatty liver disease and its relationship with liver enzymes. Genet Mol Res (2014) 13(3):5880–91. doi: 10.4238/2014.August.7.3 25117346

[53] Lee JK Shim JH Lee HC Lee SH Kim KM Lim YS. Estimation of the healthy upper limits for serum alanine aminotransferase in Asian populations with normal liver histology. Hepatology (2010) 51(5):1577–83. doi: 10.1002/hep.23505 20162730

[54] Kang HS Um SH Seo YS An H Lee KG Hyun JJ. Healthy range for serum ALT and the clinical significance of “unhealthy” normal ALT levels in the Korean population. J Gastroenterol Hepatol (2011) 26(2):292–9. doi: 10.1111/j.1440-1746.2010.06481.x 21261719

[55] Zheng MH Shi KQ Fan YC Liu WY Lin XF Li LF. Upper limits of normal for serum alanine aminotransferase levels in Chinese Han population. PloS One (2012) 7(9):e43736. doi: 10.1371/journal.pone.0043736 22962588 PMC3433469

[56] Pacifico L Ferraro F Bonci E Anania C Romaggioli S Chiesa C. Upper limit of normal for alanine aminotransferase: quo vadis? Clin Chim Acta (2013) 422:29–39. doi: 10.1016/j.cca.2013.03.030 23566931

[57] Kim BK Han KH Ahn SH. “Normal” range of alanine aminotransferase levels for Asian population. J Gastroenterol Hepatol (2011) 26(2):219–20. doi: 10.1111/j.1440-1746.2010.06603.x 21261710

[58] Liu Z Que S Xu J Peng T. Alanine aminotransferase-old biomarker and new concept: a review. Int J Med Sci (2014) 11(9):925–35. doi: 10.7150/ijms.8951 PMC4081315 25013373

[59] Schriger DL Lorber B. Lowering the cut point for impaired fasting glucose: where is the evidence? Where is the logic? Diabetes Care (2004) 27(2):592–601. doi: 10.2337/diacare.27.2.592 14747244

[60] Muntner P Carey RM Gidding S Jones DW Taler SJ Wright JT Jr. Potential US population impact of the 2017 ACC/AHA high blood pressure guideline. Circulation (2018) 137(2):109–18. doi: 10.1161/CIRCULATIONAHA.117.032582 PMC5873602 29133599

[61] Sung KC Lee MY Lee JY Lee SH Kim SH Kim SH. Utility of ALT concentration in men and women with nonalcoholic fatty liver disease: cohort study. J Clin Med (2019) 8(4):445. doi: 10.3390/jcm8040445 30987010 PMC6517922

[62] Rivera-Coll A Fuentes-Arderiu X Díez-Noguera A. Circadian rhythms of serum concentrations of 12 enzymes of clinical interest. Chronobiol Int (1993) 10(3):190–200. doi: 10.3109/07420529309073887 8100488

[63] Gnocchi D Bruscalupi G. Circadian rhythms and hormonal homeostasis: pathophysiological implications. Biol (Basel) (2017) 6(1):10. doi: 10.3390/biology6010010 PMC5372003 28165421

[64] Gnocchi D Custodero C Sabbà C Mazzocca A. Circadian rhythms: a possible new player in non-alcoholic fatty liver disease pathophysiology. J Mol Med (Berl) (2019) 97(6):741–59. doi: 10.1007/s00109-019-01780-2 30953079

[65] Joye DAM Evans JA. Sex differences in daily timekeeping and circadian clock circuits. Semin Cell Dev Biol (2022) 126:45–55. doi: 10.1016/j.semcdb.2021.04.026 33994299 PMC8589873

[66] Walton JC Bumgarner JR Nelson RJ. Sex differences in circadian rhythms. Cold Spring Harb Perspect Biol (2022) 14(7):a039107. doi: 10.1101/cshperspect.a039107 35101914 PMC9248824

[67] Bailey M Silver R. Sex differences in circadian timing systems: implications for disease. Front Neuroendocrinol (2014) 35(1):111–39. doi: 10.1016/j.yfrne.2013.11.003 PMC4041593 24287074

[68] Hatcher KM Royston SE Mahoney MM. Modulation of circadian rhythms through estrogen receptor signaling. Eur J Neurosci (2020) 51(1):217–28. doi: 10.1111/ejn.14184 30270552

[69] Krizo JA Mintz EM. Sex differences in behavioral circadian rhythms in laboratory rodents. Front Endocrinol (Lausanne) (2015) 5:234. doi: 10.3389/fendo.2014.00234 25620955 PMC4288375

[70] Talamanca L Gobet C Naef F. Sex-dimorphic and age-dependent organization of 24-hour gene expression rhythms in humans. Science (2023) 379(6631):478–83. doi: 10.1126/science.add0846 36730411

[71] Huang W Ramsey KM Marcheva B Bass J. Circadian rhythms, sleep, and metabolism. J Clin Invest (2011) 121(6):2133–41. doi: 10.1172/JCI46043 PMC3104765 21633182

[72] Pelissier AL Attolini L Gantenbein M Bruguerolle B. Tobacco smoke influence on heart rate, body temperature, and locomotor activity daily rhythms as assessed by radiotelemetry in rats. J Pharmacol Toxicol Methods (1997) 38(4):195–200. doi: 10.1016/s1056-8719(97)00102-0 9566443

[73] Saadeh S Younossi ZM Remer EM Gramlich T Ong JP Hurley M. The utility of radiological imaging in nonalcoholic fatty liver disease. Gastroenterology (2002) 123(3):745–50. doi: 10.1053/gast.2002.35354 12198701

[74] Fishbein M Castro F Cheruku S Jain S Webb B Gleason T. Hepatic MRI for fat quantitation: its relationship to fat morphology, diagnosis, and ultrasound. J Clin Gastroenterol (2005) 39(7):619–25. doi: 10.1097/00004836-200508000-00012 16000931

