# Supplementary material for: Gender perspective on the association between liver enzyme markers and non-alcoholic fatty liver disease: insights from the general population
Source: Front Endocrinol (Lausanne). 2023 Dec 6;14:1302322. doi: 10.3389/fendo.2023.1302322 (PMC10731038; doi:10.3389/fendo.2023.1302322)
Supplement: Supplementary file 1 [file Table_1.docx]

Supplementary Table 1: Collinearity diagnostics steps of ALT with other covariates.

|  | VIF | | | |  |
| --- | --- | --- | --- | --- | --- |
|  | **Step 1** | **Step 2** | **Step 3** | **Step 4** | |
| ALT | 1.2 | 1.2 | 1.2 | 1.2 | |
| Age | 1.4 | 1.4 | 1.3 | 1.3 | |
| Height | 49.7 | 1.9 | 1.4 | 1.4 | |
| Weight | 167.5 | NA | NA | NA | |
| BMI | 95.3 | 5 | 1.7 | 1.7 | |
| WC | 5.9 | 5.9 | NA | NA | |
| HDL-C | 1.8 | 1.8 | 1.8 | 1.8 | |
| TC | 1.5 | 1.5 | 1.5 | 1.4 | |
| TG | 1.7 | 1.7 | 1.7 | 1.7 | |
| FPG | 1.4 | 1.4 | 1.4 | 1.4 | |
| HbA1c | 1.2 | 1.2 | 1.2 | 1.2 | |
| SBP | 5.5 | 5.5 | 5.5 | 1.4 | |
| DBP | 5.5 | 5.5 | 5.5 | NA | |
| Exercise habits | 1 | 1 | 1 | 1 | |
| Drinking status | 1.2 | 1.2 | 1.2 | 1.1 | |
| Smoking status | 1.3 | 1.3 | 1.3 | 1.3 | |

Abbreviations: VIF: Variance inflation factor; Other abbreviations as in Table ​1.

Note: VIF = 1/(1-R^2^).

Supplementary Table 2: Collinearity diagnostics steps of AST with other covariates.

|  | VIF | | | |  |
| --- | --- | --- | --- | --- | --- |
|  | **Step 1** | **Step 2** | **Step 3** | **Step 4** | |
| AST | 1.1 | 1.1 | 1.1 | 1.1 | |
| Age | 1.4 | 1.3 | 1.3 | 1.3 | |
| Height | 49.4 | 1.9 | 1.4 | 1.4 | |
| Weight | 166.5 | NA | NA | NA | |
| BMI | 95 | 4.9 | 1.6 | 1.6 | |
| WC | 5.9 | 5.9 | NA | NA | |
| HDL-C | 1.8 | 1.8 | 1.8 | 1.8 | |
| TC | 1.4 | 1.4 | 1.4 | 1.4 | |
| TG | 1.7 | 1.7 | 1.7 | 1.7 | |
| FPG | 1.4 | 1.4 | 1.4 | 1.4 | |
| HbA1c | 1.2 | 1.2 | 1.2 | 1.2 | |
| SBP | 5.5 | 5.5 | 5.5 | 1.4 | |
| DBP | 5.5 | 5.5 | 5.5 | NA | |
| Exercise habits | 1 | 1 | 1 | 1 | |
| Drinking status | 1.2 | 1.2 | 1.2 | 1.1 | |
| Smoking status | 1.3 | 1.3 | 1.3 | 1.3 | |

Abbreviations: VIF: Variance inflation factor; Other abbreviations as in Table ​1.

Note: VIF = 1/(1-R^2^).

Supplementary Table 3: Collinearity diagnostics steps of GGT with other covariates.

|  | VIF | | | |  |
| --- | --- | --- | --- | --- | --- |
|  | **Step 1** | **Step 2** | **Step 3** | **Step 4** | |
| GGT | 1.2 | 1.2 | 1.2 | 1.2 | |
| Age | 1.4 | 1.3 | 1.3 | 1.3 | |
| Height | 49.3 | 1.9 | 1.4 | 1.4 | |
| Weight | 166.2 | NA | NA | NA | |
| BMI | 94.8 | 4.9 | 1.6 | 1.6 | |
| WC | 5.9 | 5.9 | NA | NA | |
| HDL-C | 1.8 | 1.8 | 1.8 | 1.8 | |
| TC | 1.5 | 1.5 | 1.5 | 1.4 | |
| TG | 1.7 | 1.7 | 1.7 | 1.7 | |
| FPG | 1.4 | 1.4 | 1.4 | 1.4 | |
| HbA1c | 1.2 | 1.2 | 1.2 | 1.2 | |
| SBP | 5.5 | 5.5 | 5.5 | 1.4 | |
| DBP | 5.5 | 5.5 | 5.5 | NA | |
| Exercise habits | 1 | 1 | 1 | 1 | |
| Drinking status | 1.2 | 1.2 | 1.2 | 1.1 | |
| Smoking status | 1.3 | 1.3 | 1.3 | 1.3 | |

Abbreviations: VIF: Variance inflation factor; Other abbreviations as in Table ​1.

Note: VIF = 1/(1-R^2^).
